# Tamoxifen Rechallenge Decreases Metastatic Potential but Increases Cell Viability and Clonogenicity in a Tamoxifen-Mediated Cytotoxicity-Resistant Subline of Human Breast MCF7 Cancer Cells

**DOI:** 10.3389/fcell.2020.00485

**Published:** 2020-06-30

**Authors:** Yung-Chieh Chang, Chun Hei Antonio Cheung, Yao-Lung Kuo

**Affiliations:** ^1^Institute of Basic Medical Sciences, College of Medicine, National Cheng Kung University, Tainan, Taiwan; ^2^Department of Pharmacology, College of Medicine, National Cheng Kung University, Tainan, Taiwan; ^3^Department of Surgery, National Cheng Kung University Hospital, College of Medicine, National Cheng Kung University, Tainan and Douliu, Taiwan

**Keywords:** estrogen receptor–positive, breast cancer, tamoxifen resistance, MCF (human breast cancer cell), metastatic ability

## Abstract

**Background:**

Drug resistance is frequently found in estrogen receptor–positive (ER^+^) breast cancer patients during and after prolonged tamoxifen treatment. Although tamoxifen rechallenge has been proposed for treating recurrent breast tumors, the clinical benefit of this treatment is still controversial. The aims of this study are to identify the possible tamoxifen cytotoxicity-resistant subpopulation of MCF7 cells and to determine the effects of tamoxifen rechallenge on these cells.

**Methods:**

Western blot analysis was used to determine the expression levels of various epithelial–mesenchymal transition– and cell survival/proliferation–related proteins in MCF7 and MCF7-derived, tamoxifen-mediated cytotoxicity-resistant MCF7-TAM12.5 breast cancer cells. Wound healing, Transwell migration, and invasion assays were used to examine the metastatic potential of cells. Clonogenic assays, trypan blue exclusion assays, and bromodeoxyuridine assays were used to examine clonogenicity and to determine the proliferation rate of cells.

**Results:**

We found that MCF7-TAM12.5 cells exhibited higher tolerance to tamoxifen-mediated cytotoxicity, higher metastatic potential, higher expression levels of XIAP, and lower expression levels of ERα/ERβ/HER2/Smac than MCF7 cells. In addition, MCF7 cells endogenously expressed Bcl-2α, whereas MCF7-TAM12.5 cells only expressed Bcl-2β. Interestingly, tamoxifen rechallenge decreased the metastatic potential but increased the proliferation and clonogenicity of MCF7-TAM12.5 cells. At the molecular level, tamoxifen rechallenge upregulated the expression of phosphorylated Aurora A and Aurora B kinase in MCF7-TAM12.5 cells.

**Conclusion:**

Our findings further support the existence of highly heterogenetic cancer cell populations in ER^+^ breast tumors. It will be of clinical importance to determine the protein expression and the genetic profiles of tamoxifen-resistant/recurrent ER^+^ breast tumors to predict the potential effects of tamoxifen readministration in the future.

## Introduction

Breast cancer is the second leading cause of cancer-related death among females in developed and developing countries (Siegel et al., 2019). Currently, hormonal therapy [e.g., selective estrogen receptor modulators (SERMs) and aromatase inhibitors] is the mainstay of adjuvant treatment for patients with estrogen receptor–positive (ER^+^) breast cancer (Chen and Cheung, 2018). Although tamoxifen has been widely used for the treatment of patients with ER^+^ breast cancer since 1977, resistance to this drug is frequently found in patients during and after the standard regimen duration, and recent evidence has suggested intratumor heterogeneity as one of the causes of therapeutic resistance in breast cancer (Osborne, 1998; Babayan et al., 2013; Knox et al., 2014). In fact, approximately 10–15% of breast cancer patients develop distant organ metastasis within 3 years after initial diagnosis of the primary tumor (Weigelt et al., 2005). Bone is the most frequent metastatic site in breast cancer patients. The osteotropic metastasis of breast cancer is osteolytic and driven by a vicious cycle between cancer cells and osteoclasts (Mundy, 2002; Weilbaecher et al., 2011; Ell and Kang, 2012; Mackiewicz-Wysocka et al., 2012). Breast cancer cells release some molecular factors including EGF, VEGF, CXCL12, and so on, to create a premetastatic niche and VCAM-1 and RANKL to activate osteoclasts, resulting in bone metastasis (Fontanella et al., 2015). Additionally, primary breast cancer tumors can stimulate distant premetastatic lesions by activating bone stromal cells to release numerous cytokines, chemokines, and several factors (Johnson et al., 2016). The ER^–^ breast cancer subtype has a higher rate of visceral organ metastasis than the ER^+^ breast cancer subtype (Lee et al., 2011). Therefore, it is of clinical importance to identify and characterize different populations of cells present in a tumor to further understand the mechanisms of cancer progression, hormone therapy resistance, and distant organ metastasis.

Human breast MCF7 cancer cells are commonly used for studying the biology and drug resistance development process (e.g., SERM resistance) of ER^+^ breast cancer (Comşa et al., 2015; Huang et al., 2017). In the current study, we identified a subpopulation of MCF7 cells that exhibit higher clonogenicity, metastatic potential, and cytotoxic tolerance to tamoxifen than the major MCF7 cell populations. Interestingly, we found that tamoxifen rechallenge upregulates viability and clonogenicity but decreases the metastatic potential of these cells.

## Materials and Methods

### Cell Line and Cell Culture Conditions

Human breast adenocarcinoma MCF7 cells (American Type Culture Collection, Manassas, Virginia, United States) were cultured in Dulbecco modified eagle medium (DMEM) (Bio Concept, Allschwil, Baselland, Switzerland, cat# 1-26P02-L) supplemented with 10% fetal bovine serum (FBS) (Gibco, Dún Laoghaire, Dublin, Ireland, cat# 10437-028) and penicillin–streptomycin (Gibco, Dún Laoghaire, Dublin, Ireland, cat# 15140-122). Tamoxifen-resistant MCF7-TAM12.5 cells (derived from MCF7 cells) were cultured in DMEM supplemented with 10% FBS, penicillin-streptomycin-glutamine (PSG), and 12.5 μM tamoxifen (Sigma, St. Louis, Missouri, United States, cat# T5648). All cells were cultured at 37°C in a humidified incubator containing 5% CO_2_.

### Trypan Blue Exclusion Cell Viability Assay

A total of 1 × 10^4^ cells were seeded onto 96-well plates for 24 h. The cellular viability was evaluated by mixing 100 μL of cell suspension and 100 μL of 0.4% trypan blue solution (Gibco, Dún Laoghaire, Dublin, Ireland, cat# 15250-061), and the number of cells was counted in a Neubauer chamber. The average of four readings for each sample was taken, and the cell count was calculated according to the following equation: number of cells/mL = average cell count × 2 × 10^4^.

### Bromodeoxyuridine Cell Proliferation Assay

The bromodeoxyuridine (BrdU) cell proliferation assay (Merck Millipore, Burlington, Massachusetts, United States, cat# QIA58) was used to determine the proliferation rate of cells. Briefly, cells were seeded at 3 × 10^3^/well in 96-well plates for 24 h prior to the treatments. Cells treated with or without tamoxifen were labeled with BrdU for 5 h prior to incubation with anti-BrdU monoclonal antibody for an hour. The immune complex was detected following incubation with peroxidase-conjugated anti–mouse immunoglobulin G and substrate solution. The reaction was terminated after 30 min, and the absorbance of the assay wells was quantified by measuring at 450–540 nm wavelength using the SpectraMax M5 microplate reader (Molecular Devices LLC, San Jose, California, United States). The number of proliferating cells is represented by the amount of BrdU incorporation, which directly correlates to the color intensity and the absorbance values.

### Clonogenic Assay

A total of 5 × 10^3^ MCF7 and MCF7-TAM12.5 cells were seeded onto six-well plates. MCF7-TAM12.5 cells were treated with or without 12.5 μM tamoxifen and allowed to grow for 1 week until the colonies could be observed. The colonies were fixed with 4% paraformaldehyde (Sigma-Aldrich, cat# P6158) solution for 15 min and subsequently stained with 0.2% crystal violet solution (Riedel-de Haën, Munich, Bayern, Germany, cat# 32675). The stained colonies were imaged with an inverted microscope (Nikon E400, Tokyo, Japan).

### Wound Healing Assay

A total of 2.2 × 10^4^ cells were seeded onto each well of the culture inserts (Ibidi, Gräfelfing, Germany) for 24 h. The culture inserts were removed, 500 μm cell-free gaps were created, and images of the wound areas were taken using an inverted microscope (Nikon E400) after 6, 12, and 24 h. The average width of the wound was measured and analyzed using ImageJ software (National Institutes of Health, Bethesda, MD, United States), and the migratory ability was calculated.

### Transwell Migration/Invasion Assay

For the Transwell migration assay, a total of 1 × 10^5^ cells were seeded onto the upper chamber of the Transwell (Corning, New York, United States, cat# 3464) in serum-free culture medium. For the Transwell invasion assay, the upper chambers were coated with 20% Matrigel (BD Medical Technology, Franklin Lakes, New Jersey, United States) prior to cell seeding. Cell culture medium was added to the lower chamber, and cells were incubated with or without tamoxifen for 6 h (for the Transwell migration assay) or 24 h (for the Transwell invasion assay). Then, cells attached on the reverse side of the PET membrane were fixed with 4% paraformaldehyde solution and subsequently stained with 0.2% crystal violet solution. Five random views were photographed and quantified under an inverted microscope (Nikon E400). Next, the crystal violet was dissolved with 200 μL 33% acetic acid, and the absorbance was measured (using 570 nm wavelength). The related migratory ability was calculated by comparing the absorbance intensity. Each experiment was repeated at least three times.

### Western Blotting

Cells were lysed with the CelLytic^TM^ cell lysis reagent (Sigma-Aldrich, cat# 2978) containing cocktail protease inhibitors (Roche, Basel, Kanton Basel-Stadt, Switzerland, cat# 04693159001), 1 mM phenylmethane sulfonyl fluoride, and 1 mM NaF. Equal amounts of protein were subjected to SDS-PAGE (sodium dodecyl sulfate–polyacrylamide gel electrophoresis) on a 6, 8, 10, or 12% polyacrylamide gel. Subsequently, the resolved proteins were transferred onto a polyvinylidene fluoride (PVDF) membrane (Merck Millipore, Burlington, Massachusetts, United States, cat# IPVH00010) and incubated with 5% non-fat dried milk in Tris-buffer containing 0.1% Tween 20 (TBST) for an hour at room temperature before being incubated overnight at 4°C with different primary antibodies: anti-pan actin (reacts with all six isoforms of vertebrate actin) (Merck Millipore, Burlington, Massachusetts, United States, cat# MAB 1501); anti-ERα (Genetex, Irvine, California, United States, cat# GTX70171); anti-ERβ (Santa Cruz, Dallas, Texas, United States, cat# sc373853); anti-p53 (Genetex, Irvine, California, United States, cat# GTX102965); anti-XIAP (R&D System, Minneapolis, Minnesota, United States, cat# AF8221); anti-Bcl-2 (Genetex, Irvine, California, United States, cat# 100064); anti-Smac/Diablo (Merck Millipore, Burlington, Massachusetts, United States, cat# 04-578); anti-HER2 (ERBB2) (Origene, Rockville, Maryland, UM570036); anti-MDR1 (Merck Millipore, Burlington, Massachusetts, United States, cat# MAB4120); anti-Snail (Cell Signaling, Danvers, Massachusetts, United States, cat# 3879); anti-vimentin (Genetex, Irvine, California, United States, cat#100619); anti–E-cadherin (Cell Signaling, Danvers, Massachusetts, United States, cat# 3195); anti–N-cadherin (Abcam, Cambridge, Cambridgeshire, United Kingdom, cet# ab-76011); anti–phospho-Aurora A/Aurora B/Aurora C (Cell Signaling, Danvers, Massachusetts, United States, cat# 2914); anti-Aurora A (R&D system, Minneapolis, Minnesota, United States, cat# AF3295); and anti–Aurora B (Genetex, Irvine, California, United States, cat# GTX130211). The PVDF membrane was then washed with TBST before incubation for 1 h at room temperature with different peroxidase-conjugated secondary antibodies. Immune complexes were finally detected with chemiluminescence reagents, and luminescence protein signals were detected by luminescence readers (FUJILAS-100; Fujifilm, Minato, Tokyo, Japan). The intensity of bands was analyzed by ImageJ software (National Institutes of Health).

### Statistical Analysis

Each experiment was repeated at least three times (i.e., three to four times). Data are presented as the mean ± s.d. (standard deviation). The significance of differences was evaluated with one-way analysis of variance (parametric). *P* < 0.05 was considered statistically significant.

## Results

### Molecular Characterizations of a Subpopulation of MCF7 Cancer Cells That Exhibit Reduced Therapeutic Sensitivity to Tamoxifen

**FIGURE 1:**
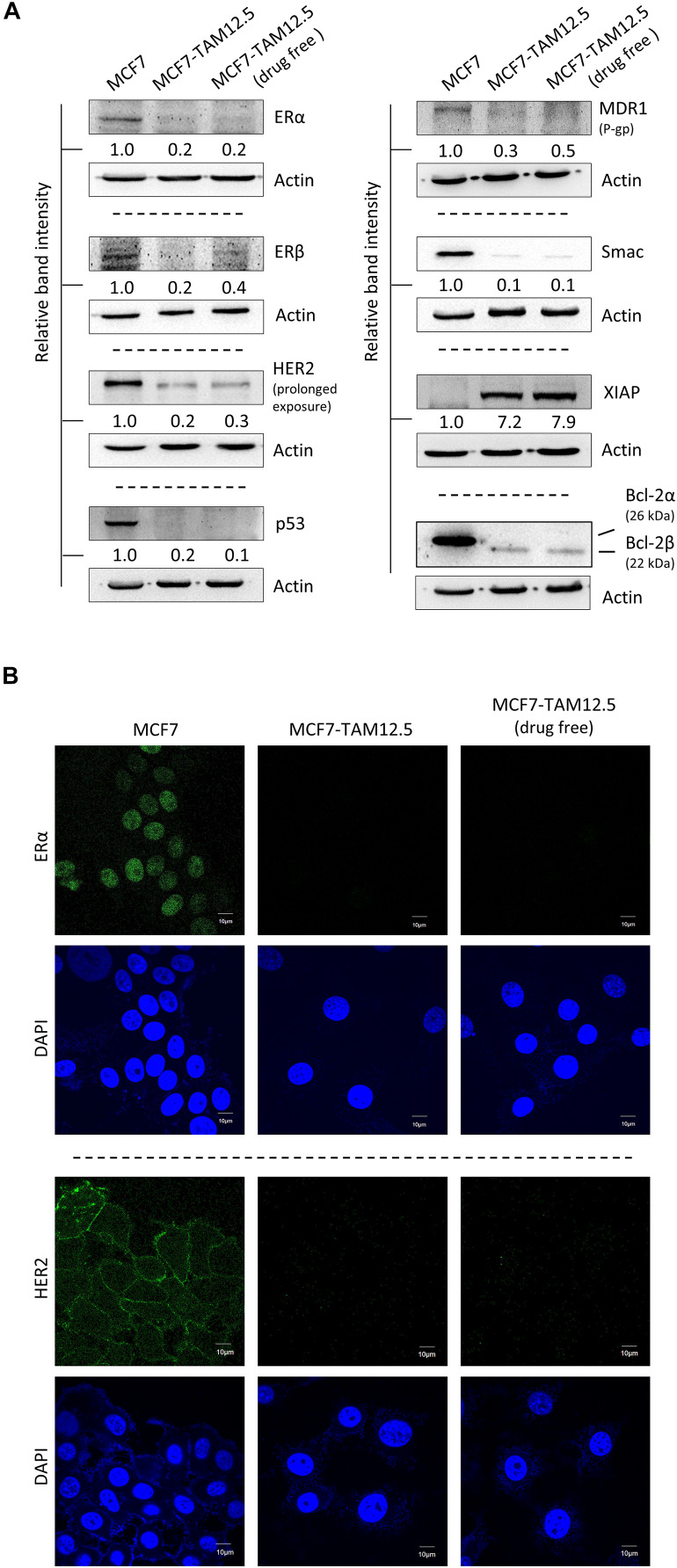
Molecular characterizations of MCF7 and MCF7-TAM12.5 cells. **(A)** Protein expression level of ERα, ERβ, HER2, p53, MDR1, Smac, XIAP, and Bcl-2 was analyzed in MCF7, MCF7-TAM12.5 (under 12.5 μM tamoxifen), and MCF7-TAM12.5 (drug free) cells by Western blotting. Equal protein loading was verified by actin. The numbers under each blot are the intensities of the blot relative to MCF7 cells. **(B)** Location of ERα and HER2 (green fluorescence) was visualized by immunofluorescence microscopy. Nuclei were counterstained blue by DAPI.

The human breast cancer cell line MCF7 was originally thought to be a monoclonal cell line but were recently discovered as populations of breast cancer cells with high levels of molecular heterogeneity (but mostly ER^+^, wild-type p53^+^, estrogen-dependent, and tamoxifen-sensitive) (Leung et al., 2010, 2014). In the current study, we identified a subpopulation of MCF7 cells, namely, MCF7-TAM12.5 cells, which are capable of surviving in medium containing 12.5 μM tamoxifen (i.e., IC_50_ in MCF7 cells in terms of cell viability). Downregulation of ER is known to promote tamoxifen or hormone therapy resistance in ER^+^ breast cancer. Here, molecular analysis revealed that MCF7-TAM12.5 cells exhibit lower expression of ERα and ERβ (i.e., ERα^low^/β^low^) than MCF7 cells regardless of the presence of tamoxifen (12.5 μM) (Figures 1A,B). In addition, MCF7-TAM12.5 cells do not express (or express but at an undetectable level) the well-known tumor suppressor p53 (Figure 1A).

Smac is a proapoptotic molecule that can bind to the antiapoptotic molecule XIAP and subsequently promote the degradation of XIAP. In contrast, Bcl-2β is a splice variant of Bcl-2α (i.e., the wild-type Bcl-2), and overexpression of this Bcl-2 isoform has been shown to inhibit apoptosis and to increase chemoresistance/UV resistance in cancer cells (Schinkothe et al., 2006; Warren et al., 2016). As shown in Figure 1A, compared to the parental cell line, MCF7-TAM12.5 cells exhibited Smac downregulation (i.e., Smac^low^) and Bcl-2α depletion (i.e., Bcl-2α^–^), but XIAP upregulation (i.e., XIAP^hi^) and Bcl-2β expression (i.e., Bcl-2β^+^) (Figure 1A).

Upregulation of human epidermal growth factor receptor 2 (HER2) is frequently found in tamoxifen-resistant or estrogen-independent ER^+^ breast cancer. Surprisingly, compared to MCF7 cells, MCF7-TAM12.5 cells show decreased expression of HER2 (i.e., HER2^low^) and multidrug resistance protein (i.e., MDR1^low^), which is a well-known multidrug efflux pump, indicating that MCF7-TAM12.5 cells induce tamoxifen resistance mostly through a HER2- and MDR1-independent mechanism (Figures 1A,B).

### Tamoxifen-Treated Breast Cancer Patients With High XIAP Expression Levels Show Poor Prognostic Outcomes

**FIGURE 2:**
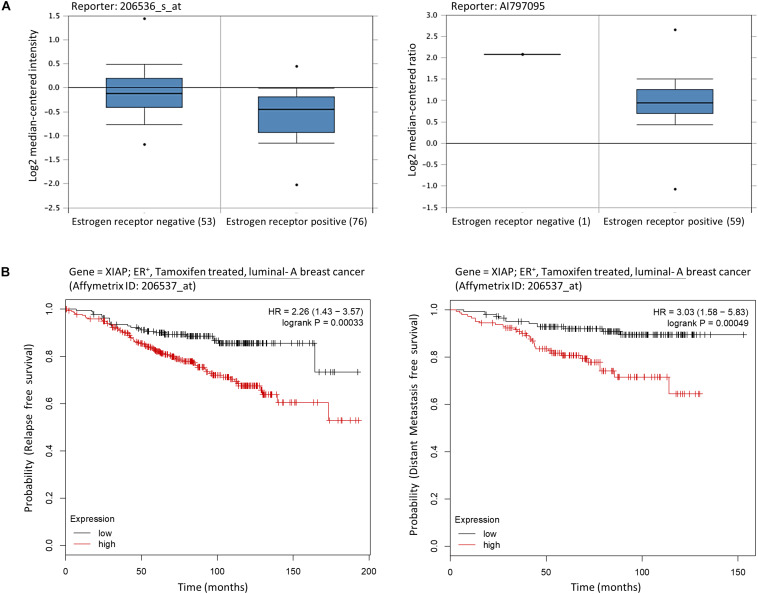
High XIAP mRNA expression level is positively correlated with poor survival of breast cancer patients. **(A)** The mRNA expression levels of XIAP in normal and tumor tissues of patients with estrogen receptor–negative and estrogen receptor–positive breast cancer were analyzed using the database and software available online (Oncomine^TM^) (see text footnote 1). **(B)** Kaplan–Meier survival estimates of high (red line) or low (black line) XIAP expression in ER^+^ tamoxifen-treated luminal A breast cancer subtype. Analysis was performed using the online database and web tool (Kaplan–Meier plotter, https://kmplot.com/).

As mentioned previously, MCF7-TAM12.5 cells exhibit reduced expression of ERα/β and increased expression of XIAP compared to MCF7 cells. Intriguingly, assessments of mRNA expression profiles derived from clinical samples of breast cancer patients using a database available online (Oncomine^TM^)^1^ revealed that the amount of XIAP mRNA transcripts present in ER^–^ breast tumor tissues is significantly higher than that in ER^+^ breast tissues in different cohorts (Figure 2A). In addition, Kaplan–Meier analysis of expression cohorts of ER^+^ breast tumors showed that high XIAP expression levels significantly (*p* < 0.0001) correlate with poor relapse-free survival [hazard ratio (HR) > 2] and poor distant metastasis-free survival (HR > 3) in tamoxifen-treated ER^+^ (luminal-A) breast cancer patients (Figure 2B). These results suggest that XIAP upregulation might in part contribute to the therapeutic tolerance to tamoxifen found in MCF7-TAM12.5 cells.

### Tamoxifen Decreases the Migration and Invasion Ability of MCF7-TAM12.5 Cells

**FIGURE 3:**
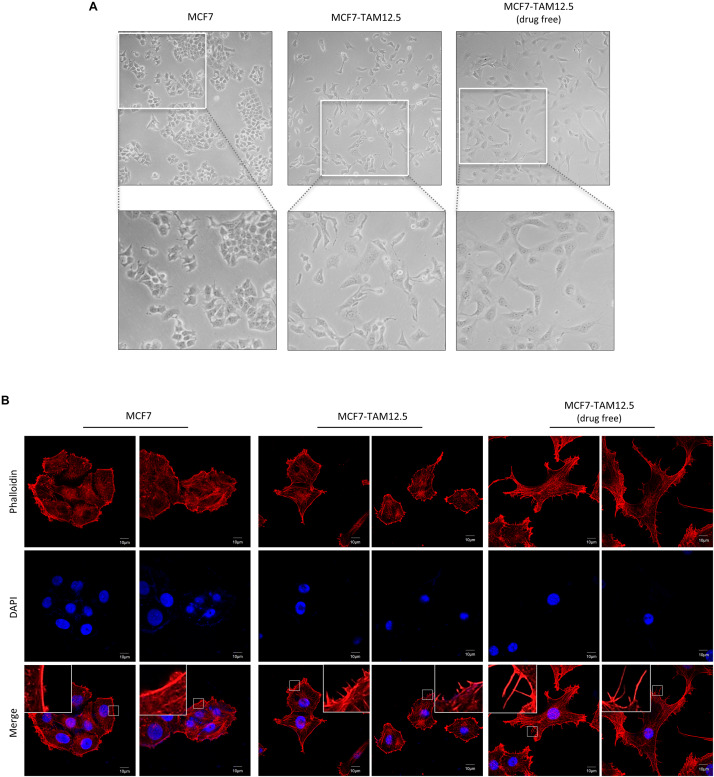
MCF7-TAM12.5 cells exhibit mesenchymal cell–like morphology. **(A)** The general morphology of MCF7, MCF7-TAM12.5 (under 12.5 μM tamoxifen), and MCF7-TAM12.5 (drug free) cells was observed under the light microscope (i.e., bright-field microscopy). **(B)** The cytoskeleton network of MCF7, MCF7-TAM12.5 (under 12.5 μM tamoxifen), and MCF7-TAM12.5 (drug free) cells was visualized by staining with phalloidin. Nuclei were counterstained blue by DAPI.

We sought to further characterize MCF7-TAM12.5 cancer cells at the cellular level. In culture, most MCF7 cells had a rounded cell shape, whereas MCF7-TAM12.5 cells displayed an elongated and mesenchymal cell–like morphology (Figure 3A). In addition, cytoskeleton analysis (by actin staining) revealed that MCF7-TAM12.5 cells contained more lamellipodium extensions than MCF7 cells (Figure 3B). Intriguingly, tamoxifen removal further increased the numbers and length of lamellipodium extensions in MCF7-TAM12.5 cells, indicating that MCF7-TAM12.5 cells might have an increased metastatic potential compared to that in MCF7 cells and that the use of tamoxifen might be able to reduce this potential in MCF7-TAM12.5 cells (Figure 3B, right panels).

**FIGURE 4:**
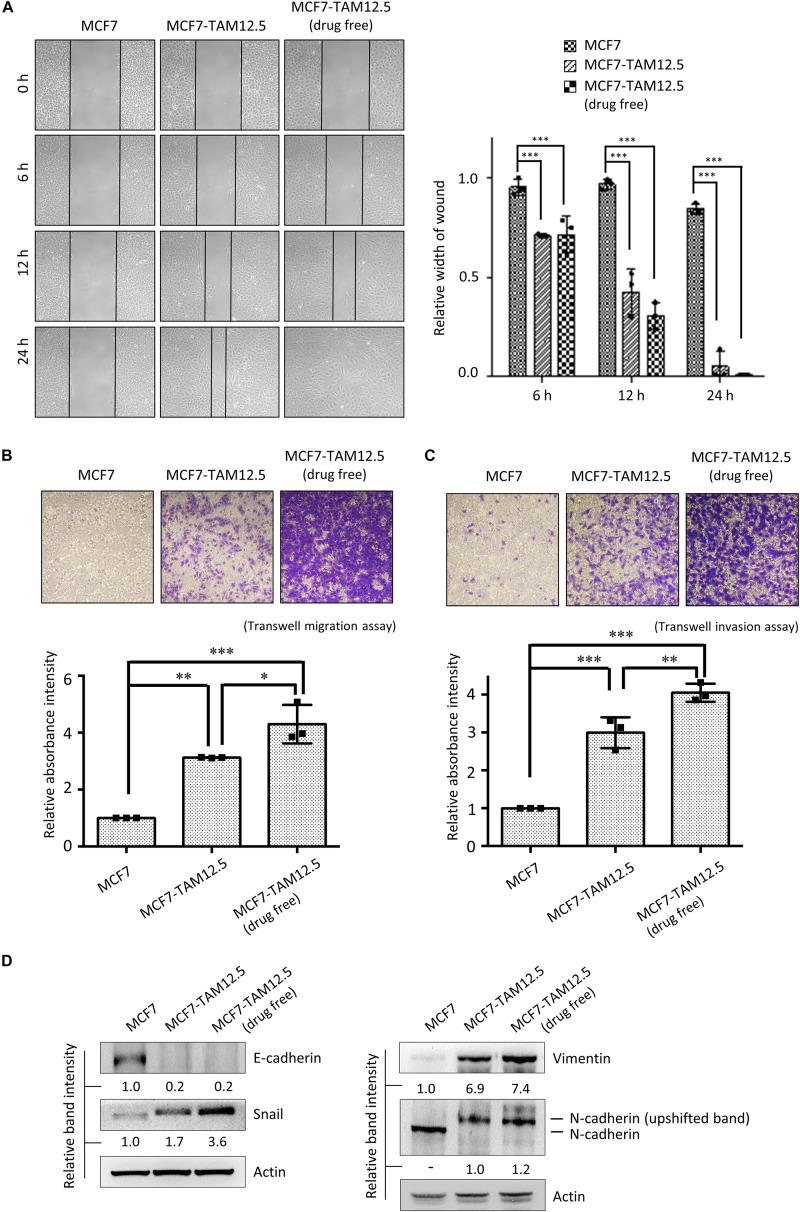
Tamoxifen decreases the migration and invasion ability of MCF7-TAM12.5 cells. **(A)** The migration ability of MCF7, MCF7-TAM12.5 (under 12.5 μM tamoxifen), and MCF7-TAM12.5 (drug free) cells was analyzed using the wound healing assay. The width of gap was measured by the ImageJ software. A statistically significant difference of relative width of gap comparing between the subpopulation MCF7 cells is denoted by ^∗∗∗^ (*p* < 0.001). **(B,C)** The migration/invasion ability of MCF7, MCF7-TAM12.5 (under 12.5 μM tamoxifen), and MCF7-TAM12.5 (drug free) cells was analyzed using the Transwell migration and invasion assay. Microscopic images were taken 6 h (for the migration assay) and 24 h (for the invasion assay) after initiation. A statistically significant difference of relative absorbance intensity comparing between the testing groups is denoted by ^∗^ (*p* < 0.05), ^∗∗^ (*p* < 0.01), ^∗∗∗^ (*p* < 0.001). **(D)** The expression of E-cadherin, Snail, vimentin, and N-cadherin was analyzed by Western blotting. Equal protein loading was verified by actin. The numbers under each blot are the intensities of the blot relative to MCF7 cells.

The migration and invasion ability of MCF7 and MCF7-TAM12.5 (with or without tamoxifen coincubation) cells was subsequently examined by using wound healing and Transwell cell migration/invasion assays. In alignment with the cell morphologies, MCF7-TAM12.5 cells (under normal culturing conditions, i.e., contained 12.5 μM tamoxifen) clearly exhibited higher migration and invasion ability than MCF7 cells. Noticeably, the migration and invasion ability of MCF7-TAM12.5 cells was further increased under tamoxifen-free conditions (Figures 4A–C).

Consistent with the results of the cell migration and invasion assays, compared to MCF7 cells, MCF7-TAM12.5 cells (under normal culturing conditions) showed increased expression of Snail and vimentin but decreased expression of E-cadherin, which are typical molecular changes in cells with EMT-like phenotypes (Figure 4D; Davidson et al., 2012). Overexpression, phosphorylation, and membrane translation of N-cadherin have been demonstrated in cells with increased migration and invasion ability (Qi et al., 2006; Shih and Yamada, 2012). Interestingly, the results of the Western blot analysis showed the presence of the possible phosphor-N-cadherin molecule (i.e., upshifted the N-cadherin band) in MCF7-TAM12.5 cells but not in MCF7 cells (or mainly found in MCF7-TAM12.5 cells) (Figure 4D). Noticeably, the expression of the EMT markers, Snail and vimentin, was further increased in MCF7-TAM12.5 cells under tamoxifen-free culture conditions (Figure 4D). The expression of the “possible phosphor-N-cadherin molecule” was also slightly increased in MCF7-TAM12.5 cells under the same conditions (Figure 4D). Collectively, these results suggest that even though tamoxifen is incapable of promoting the death of MCF7-TAM12.5 cells, it can lower the migration and invasion ability of these cancer cells.

### Tamoxifen Promotes the Proliferation and Increases the Clonogenicity of MCF7-TAM12.5 Cells

**FIGURE 5:**
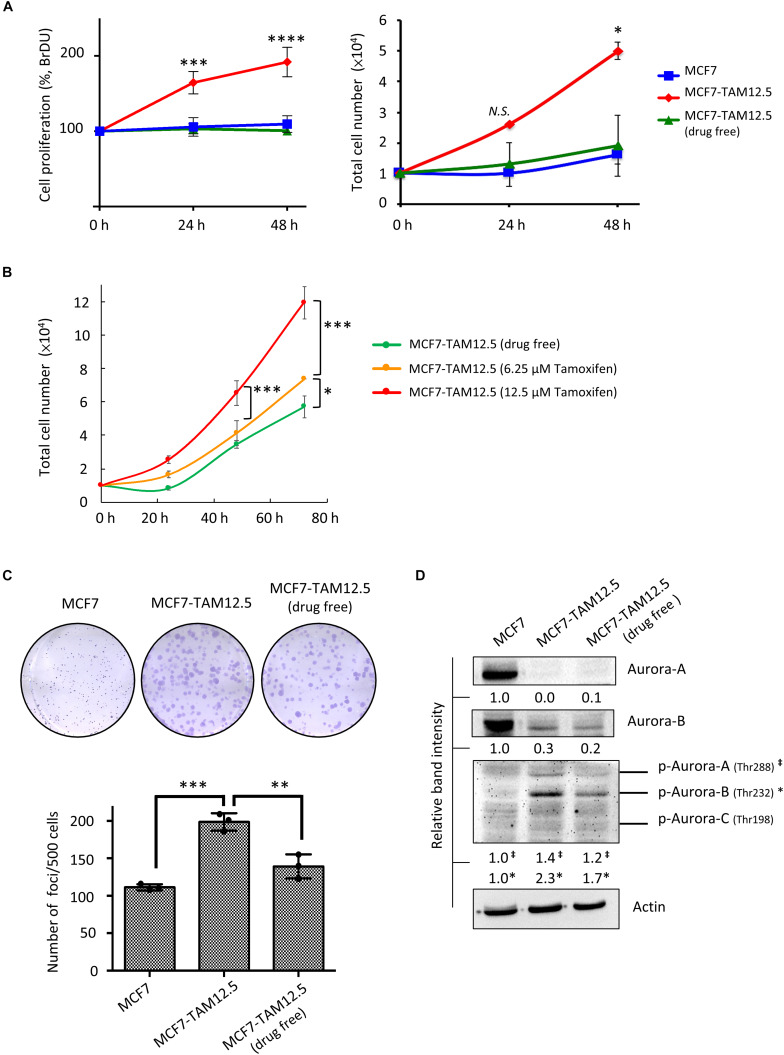
Tamoxifen promotes the proliferation and clonogenicity of MCF7-TAM12.5 cells. **(A)** The proliferation of MCF7 and MCF7-TAM12.5 cells with or without 12.5 μM tamoxifen treatment was analyzed using the BrdU cell proliferation assay (left) and trypan blue exclusion cell viability assay (right) 24 and 48 h post-treatments. A statistically significant difference of cell proliferation between MCF7-TAM12.5 cells and MCF7 cells is denoted by ^∗^ (*p* < 0.05), ^∗∗∗^ (*p* < 0.001), ^****^ (*p* < 0.0001). N.S. denotes no statistical significance difference between the testing groups. **(B)** The proliferation of MCF7-TAM12.5 cells with or without tamoxifen treatment was analyzed using the trypan blue exclusion cell viability assays. **(C)** Clonogenicity of MCF7, MCF7-TAM12.5 (under 12.5 μM tamoxifen), and MCF7-TAM12.5 (drug free) cells was analyzed using the clonogenic assay. A statistically significant difference in the number of colony formation between the testing groups is denoted by ^∗∗^ (*p* < 0.01), ^∗∗∗^ (*p* < 0.001). **(D)** The expression of different proteins in MCF7, MCF7-TAM12.5 (under 12.5 μM tamoxifen), and MCF7-TAM12.5 (drug free) cells was determined by Western blotting. Equal protein loading was verified by actin. The numbers under each blot are the intensities of the blot relative to MCF7 cells.

The growth rate of MCF7-TAM12.5 cells under tamoxifen-free conditions was similar to that of MCF7 cells (Figure 5A). Surprisingly, although tamoxifen decreased the metastatic potential of MCF7-TAM12.5 cells, the results of the BrdU cell proliferation assay and the trypan blue exclusion cell viability assay showed that tamoxifen significantly promoted the growth of MCF7-TAM12.5 cells in a concentration-dependent manner (Figures 5A,B). The doubling times for MCF7, MCF7-TAM12.5 (with tamoxifen), and MCF7-TAM12.5 (without tamoxifen) cells were 36, 17, and 48 h, respectively. The results of the clonogenic assay (i.e., colony formation assay) showed that MCF7-TAM12.5 cells exhibited higher clonogenicity than MCF7 cells (Figure 5C). Similar to the results of the cell proliferation assay, the presence of tamoxifen further increased the clonogenicity of MCF7-TAM12.5 cells *in vitro* (Figure 5C).

Aurora A and B kinases are known to play important roles in cell division, especially during G_2_/M phase (Hálová and Petersen, 2011; Gully et al., 2012). As shown in Figure 5D, compared to MCF7 cells, MCF7-TAM12.5 cells exhibited increased expression of phosphorylated Aurora A and B kinase (especially phosphorylated Aurora B kinase) under tamoxifen-free culture conditions. Again, the presence of tamoxifen further increased the expression of phosphorylated Aurora A and B kinase in MCF7-TAM12.5 cells (Figure 5D). Taken together, these results reveal that tamoxifen increases the proliferation and clonogenicity of MCF7-TAM12.5 cells.

## Discussion

Human breast MCF7 cancer cells are commonly used for studying the biology and drug resistance development process of ER^+^ (or luminal A subtype-like) breast cancer (Comşa et al., 2015). Although MCF7 was originally thought to be a monoclonal cell line, it was recently discovered as a population of breast cancer cells (mostly ER^+^) with high levels of molecular heterogeneity (Leung et al., 2010; Pérez-Yépez et al., 2012; Khan et al., 2017; Roden et al., 2018). In this study, we identified a subpopulation (or a subline) of MCF7 cells (i.e., the ERα^low^/β^low^-p53^–/low^-HER2^low^-Smac^low^-XIAP^hi^-Bcl-2β^+^ MCF7-TAM12.5 cells), which exhibit higher therapeutic tolerance (i.e., cell death resistance, during primary challenge) and have differential metastatic potential and clonogenic responses (upon rechallenge) against tamoxifen.

It is widely accepted that the hormone therapeutic agent tamoxifen induces ER^+^ breast cancer cell death by interfering with the interactions between estrogen and ERs. In addition, it has been demonstrated that p53 status influences the response to tamoxifen in breast cancer cells (Fernandez-Cuesta et al., 2011). Thus, it was not surprising to see that MCF7-TAM12.5 cells exhibit reduced expression of ERα, ERβ, and p53. However, it is surprising to see that p53^–^/p53^low^ MCF7-TAM12.5 cells exhibit lower expression of HER2 than MCF7 cells. Upregulation of HER2 in the tamoxifen-resistant, ER^+^, MCF7 sublines has previously been reported in different studies, and upregulation of this hormone receptor is also known to promote tamoxifen/endocrine resistance in ER^+^ breast cancer (Benz et al., 1992; Arpino et al., 2004; Huang et al., 2017). In fact, it has been shown that the cross-talk between ERα, HER2, and EGFR plays an important role in tamoxifen-induced proliferation in p53-mutated (or downregulated) breast cancer cells (Fernandez-Cuesta et al., 2011). Nevertheless, the results of our molecular analysis suggest that MCF7-TAM12.5 cells increase their therapeutic (i.e., cytotoxic) tolerance against tamoxifen, possibly through a HER2-independent mechanism. Of note, we also observed expression of Bcl-2β [i.e., a splice variant of wild-type Bcl-2 (Bcl-2α)] in MCF7-TAM12.5 cells. Since the discovery of Bcl-2β in 2006, few studies have reported the existence of this Bcl-2 isoform in cells (Schinkothe et al., 2006; Warren et al., 2016). Therefore, it is surprising to find that MCF7-TAM12.5 cells (i.e., a subpopulation of MCF7 cells) express Bcl-2β, whereas MCF7 cells (i.e., the major populations) only express Bcl-2α. At the molecular level, Bcl-2β binds to a variety of proapoptotic proteins, such as Bad, Bax, and Bid. Although Schinkothe et al. (2006) demonstrated that Bcl-2β exhibits higher antiapoptotic activity than Bcl-2α, it is still unclear whether the expression of Bcl-2β plays a role in the therapeutic tolerance against tamoxifen in MCF7-TAM12.5 cells, considering that the expression levels of Bcl-2α and Bcl-2β in MCF7 and MCF7-TAM12.5 cells are very different. It is worth noting that MCF7-TAM12.5 cells overexpress Snail and vimentin, which are molecular markers for cellular EMT. Interestingly, Jiang et al. (2014) demonstrated that ectopic expression of Snail induced tamoxifen resistance in ER^+^ breast cancer cells *via* an EMT-independent mechanism. Therefore, the overexpression of Snail might partially contribute to the induction of tamoxifen resistance (i.e., therapeutic resistance) in MCF7-TAM12.5 cells.

Smac is a well-known proapoptotic molecule that promotes cell death in part through physical interactions with different members of the inhibitor-of-apoptosis (IAP) protein family, such as XIAP and survivin (Cheung et al., 2020). For example, the binding of Smac to XIAP inhibits the physical interactions between XIAP and caspase-9, leading to the activation of caspase-9 and apoptosis (Srinivasula et al., 2001). Of note, a study by Zhao et al. (2014) reported that anthracycline neoadjuvant chemotherapy–treated breast cancer patients with high expression of Smac and low expression of survivin had longer disease-free survival and overall survival than those with low expression of Smac and high expression of survivin (Zhao et al., 2014). In addition, overexpression of Smac (or treatment with Smac peptide) was shown to enhance apoptosis induced by tamoxifen in breast cancer cells (Fandy et al., 2008). Inhibition of XIAP by the small molecule inhibitor embelin was also shown to reduce the proliferation and migration of MCF7 cells *in vitro* (Sumalatha et al., 2014). Therefore, the reduced expression of Smac (and the increased expression of its regulating antiapoptotic molecule, XIAP) may play a role in the induction therapeutic tolerance against tamoxifen in MCF7-TAM12.5 cells. As various Smac mimetics have recently been developed, and their therapeutic potentials are currently being evaluated in patients with different cancer types (e.g., Clinicaltrials.gov ID: NCT03111992 and NCT02890069) (Chen et al., 2018; Yang et al., 2019), the findings of this study further support the clinical assessment of different Smac mimetics in patients with tamoxifen-resistant breast cancer.

At the cell behavioral level, we found in the current study that tamoxifen rechallenge decreases the metastatic potential but increases the proliferation and clonogenicity of MCF7-TAM12.5 cells (even though the metastatic potential of the rechallenged MCF7-TAM12.5 cells is still higher than the untreated MCF7 cells). A previous study reported the dependence of one of the MCF7 cancer cell–derived xenografts on tamoxifen for survival and proliferation; this xenograft showed tamoxifen resistance during primary treatment after retransplantation (Gottardis and Jordan, 1988). Notably, the reported “ER^+^, tamoxifen-dependent, MCF7-derived subline” was found to exhibit ER expression levels similar to those of the parental MCF7 cells (Gottardis and Jordan, 1988). Tangkeangsirisin et al. (2004) also showed that tamoxifen promoted the growth of ER^+^ MCF7 cells ectopically overexpressing PCDGF/GP88 *in vivo*. In contrast to these studies, our examined MCF7-TAM12.5 cells showed a significant reduction in the expression of ERα and ERβ. Moreover, the ERα^low^/β^low^ MCF7-TAM12.5 cells clearly survived independent of the presence of tamoxifen. It is still unclear why tamoxifen rechallenge limits the metastatic potential (to a certain extent) but increases the proliferation and clonogenicity of MCF7-TAM12.5 cells. In fact, tamoxifen is known to modulate multiple signaling pathways in cancer cells. For example, 4-OHT (an active metabolite of tamoxifen) treatment has been shown to induce the production of extracellular insulin-like growth factor–binding protein-1, partially contributing to the inhibition of insulin-like growth factor 1 (IGF-1)–dependent cell signaling in MCF-7 cells (Pratt and Pollak, 1993; Vaziri-Gohar and Houston, 2016). Notably, hyperactivation of membrane growth factor receptors, such as IGF-IR and EGFR, is known to promote the development of hormone resistance in breast cancer (Song et al., 2007). Further studies are needed to better understand the mechanisms behind the differential effects of tamoxifen in ERα^low^/β^low^/HER2^low^ breast cancer cell populations (e.g., MCF7-TAM12.5).

## Conclusion

The clinical benefit of tamoxifen rechallenge (i.e., readministration) in patients with recurrent breast cancer is still unclear and remains controversial. The findings of the current study reveal that tamoxifen rechallenge may promote proliferation and clonogenicity but decrease metastatic potential in a group of cancer cells, which are present in the ER^+^ breast tumor mass and exhibit higher cytotoxic tolerance to tamoxifen (i.e., primary challenge). Thus, it will be important to determine the protein expression and the genetic profiles of tamoxifen-resistant/recurrent breast tumors before tamoxifen readministration in the future.

## Data Availability Statement

The datasets for this article are not publicly available. Requests to access the datasets should be directed to Y-LK, ylkuo@mail.ncku.edu.tw.

## Ethics Statement

The study was approved by the institutional review board of the National Cheng Kung University Hospital (IRB No. A-ER-105-471). The patients/participants provided their written informed consent to participate in this study.

## Author Contributions

Y-LK and CC designed the study, analyzed the data, and wrote the manuscript with input from all authors. Y-LK and Y-CC performed the experiments. All authors contributed to the article and approved the submitted version.

## Conflict of Interest

The authors declare that the research was conducted in the absence of any commercial or financial relationships that could be construed as a potential conflict of interest.

## Footnotes

1 https://www.oncomine.org
